# Identification of diagnostic and therapeutic biomarkers for attention‐deficit/hyperactivity disorder

**DOI:** 10.1002/kjm2.12931

**Published:** 2025-01-07

**Authors:** Sheng‐Yu Lee, Liang‐Jen Wang, Cheng‐Fang Yen

**Affiliations:** ^1^ Department of Psychiatry Kaohsiung Veterans General Hospital Kaohsiung Taiwan; ^2^ Department of Psychiatry, School of Medicine Kaohsiung Medical University Kaohsiung Taiwan; ^3^ Department of Child and Adolescent Psychiatry Kaohsiung Chang Gung Memorial Hospital and Chang Gung University College of Medicine Kaohsiung Taiwan; ^4^ Institute for Translational Research in Biomedicine Kaohsiung Chang Gung Memorial Hospital Kaohsiung Taiwan; ^5^ Department of Psychiatry Kaohsiung Medical University Hospital, Kaohsiung Medical University Kaohsiung Taiwan; ^6^ College of Professional Studies National Pingtung University of Science and Technology Pingtung Taiwan

**Keywords:** attention‐deficit/hyperactivity disorder (ADHD), biomarker, microbiota, miRNA, neuroendocrine

## Abstract

Attention‐deficit/hyperactivity disorder (ADHD) is a common psychiatric condition among children and adolescents, often associated with a high risk of psychiatric comorbidities. Currently, ADHD diagnosis relies exclusively on clinical presentation and patient history, underscoring the need for clinically relevant, reliable, and objective biomarkers. Such biomarkers may enable earlier diagnosis and lead to improved treatment outcomes. Our research team has focused on identifying potential biomarkers for ADHD by investigating its possible pathomechanisms, with consideration of the aforementioned criteria. Given the significant sex‐related differences in ADHD prevalence (male predominance) and the age‐related variability in its symptomatology, we explored the role of neuroendocrine systems in ADHD. Specifically, we examined the epigenetic regulation mechanism involved in ADHD pathogenesis and developed a diagnostic model based on peripheral microRNA. Additionally, we investigated the role of microbiota dysbiosis in the pathophysiology of ADHD and provided novel insights into its management. This paper presents a summary of our findings on potential biomarkers for ADHD. By analyzing blood, salivary, and fecal samples, we identified several promising biomarkers that may serve as objective parameters for improving the diagnostic accuracy for ADHD. Further research involving larger cohort studies is required to confirm the reliability of these biomarkers.

## 1 INTRODUCTION

Attention‐deficit/hyperactivity disorder (ADHD) is a common psychiatric disorder among children and adolescents, affecting 5%–10% of school‐age children worldwide. The core symptoms of ADHD are inattention, hyperactivity, and impulsivity. These symptoms can hinder progress in key developmental areas, including intellectual growth, interpersonal relationships, physical and mental health, and academic performance. Evidence from clinical and epidemiological studies indicates that children with ADHD are typically at an increased risk of developing psychiatric comorbidities later in life.^1^ Early detection and accurate diagnosis are therefore essential for enabling timely interventions, which can mitigate functional impairments and improve outcomes.^2^

Currently, diagnosing ADHD exclusively relies on each individual's clinical presentation and history, as outlined in the *Diagnostic and Statistical Manual of Mental Disorders, Fifth Edition*.^3^ However, this approach presents several challenges: identifying individuals at risk, rapidly and accurately establishing a diagnosis, distinguishing between different subtypes of ADHD for optimal treatment and management, and reliably evaluating the severity of symptoms. Identifying clinically relevant, reliable, and objective biomarkers for ADHD may facilitate earlier diagnosis and lead to improved treatment outcomes. Because of the clinical heterogeneity of ADHD, utilizing such biomarkers may lead to a more refined classification of the disorder.

Although many potential biomarkers have been identified for ADHD,^4^, ^5^ the exact etiology of the disorder remains unknown.^6^ Over the past decade, our research team has focused on investigating the pathogenesis of ADHD with two primary objectives. These objectives are to identify potential biomarkers playing a key role in the pathophysiology of ADHD and to facilitate the development of novel therapeutic strategies and management approaches for ADHD.

A biomarker is an indicator that reflects the pathogenetic processes of a disorder. According to Thome et al.,^6^ an optimal ADHD biomarker must meet several criteria: high diagnostic sensitivity and specificity, noninvasiveness, reliability, reproducibility, cost‐effectiveness, and simplicity in measurement with consistent results. Guided by these criteria, our research team has endeavored to identify biomarkers for ADHD on the basis of its potential pathomechanisms. Given the male‐dominant prevalence of ADHD and its symptom variability across age, we investigated the role of the neuroendocrine system in ADHD. Recognizing the complexity of ADHD pathogenesis and the potential role of epigenetic processes in neurodevelopment and ADHD risk, we explored the role of epigenetic regulation in the pathogenesis of ADHD. Additionally, because the gut microbiota's influence on brain function and behavior has led to the concept of the “gut–brain axis,” we investigated microbiota dysbiosis to gain insights into the pathophysiology of ADHD and provided novel insights that may improve its management. In the following sections, we present a summary of our findings on potential biomarkers for ADHD.

## 2 POTENTIAL BIOMARKERS

### 2.1 Neuroendocrine systems

#### 2.1.1 Sex‐related hormones: dehydroepiandrosterone sulfate, sex hormone‐binding globulin, and steroid sulfatase gene

Epidemiological evidence suggests an ADHD prevalence of 3%–7% among school‐age children,^7^ with a male‐dominant prevalence and a boy‐to‐girl prevalence ratio ranging from 2:1 to 9:1.^8^ These sex‐related differences suggest a potential role for the neuroendocrine system in the etiology of ADHD.^9^ The neuroendocrine system, the activation of which is closely associated with age and sex, may influence the development of neural circuitry and behavioral systems. Dehydroepiandrosterone sulfate (DHEA‐S), the sulfated form of dehydroepiandrosterone (DHEA), is the most abundant steroid in the human body,^10^ produced by the adrenal cortex and the brain.^11^ In our previous study, patients with ADHD exhibited lower DHEA‐S levels compared with their healthy male and female counterparts. In addition, DHEA‐S levels were negatively correlated with children's impulsivity performance in Conners' Continuous Performance Test (CPT). Moreover, sex hormone‐binding globulin (SHBG), a protein regulating the bioavailability of sex hormones, was negatively correlated with ADHD behavioral symptoms, but only in boys.^12^ The steroid sulfatase (*STS*) gene plays a role in androgen synthesis and metabolism.^13^ Its deficiency has been proposed as a risk factor for ADHD.^14^, ^15^ Studies suggest that the X‐linked gene *STS* may influence attentional function and motor impulsivity through its interaction with DHEA‐S.^16^ Our previous study revealed that *STS* polymorphisms were associated with high DHEA‐S levels in boys with ADHD, potentially linking the gene to the neurobiological pathogenesis of ADHD.^17^

Methylphenidate (MPH), a psychostimulant, is the most commonly prescribed medication for managing ADHD in children. However, the effect of long‐term MPH treatment on hormone indices in children with ADHD remains debated. Our previous longitudinal study revealed that, after 1 year of low‐dose MPH treatment, no significant changes in thyroid or growth hormones were observed.^18^ Additionally, usual doses of MPH did not significantly alter gonadal function trends, including SHBG, follicle‐stimulating hormone (FSH), luteinizing hormone (LH), estradiol, progesterone, testosterone, free testosterone, and prolactin, in patients with ADHD over the course of 1 year.^18^ These findings have practical implications for clinicians and patients with ADHD: long‐term MPH treatment at standard doses does not significantly affect hormone trends or pubescent development in patients with ADHD.^19^

#### 2.1.2 Appetite hormones

Multiple studies have explored the correlation between appetite hormones and neuropsychiatric disorders.^20^ This growing interest stems from the understanding that appetite hormones not only regulate metabolic processes but also influence neuronal excitability and synaptic plasticity, thereby affecting cognitive function, mood, and brain development.^21^ In our previous study, we examined the role of appetite hormones in ADHD. We recruited three groups of children: unmedicated children with ADHD (ADHD‐MPH group), children with ADHD who received MPH treatment (ADHD+MPH group), and 87 healthy controls (HCs). Our results revealed that the levels of orexin in the control group were significantly higher than those in both the ADHD‐MPH and ADHD+MPH groups. In addition, the levels of orexin were significantly higher in the ADHD‐MPH group than in the ADHD+MPH group. Moreover, the levels of leptin in both the ADHD+MPH and ADHD‐MPH groups were significantly lower than those in the control group. The levels of ghrelin were positively associated with auditory attention across all ADHD groups. They were also positively correlated with MPH dosage but negatively correlated with the side effects of MPH. Taken together, these findings provide valuable insights into the relationships between appetite hormones, pharmacotherapy, and ADHD. Orexin A and leptin are associated with the etiology of ADHD, whereas orexin A and ghrelin play a crucial role in attention deficits and the need for MPH for ADHD management.^22^

#### 2.1.3 Endocrine‐disrupting chemicals

In our previous study, we explored the potential impact of exposure to endocrine‐disrupting chemicals (EDCs), such as phthalates, *para*‐hydroxybenzoic acids, and bisphenol‐A, on gonadal hormones and their potential association with increased susceptibility to ADHD. We reported that boys with ADHD had significantly higher levels of mono‐*n*‐butyl phthalate and ethylparaben compared with the control group. However, no significant differences in EDC levels were observed between girls with ADHD and the control group. Additionally, no significant differences in the levels of testosterone, free testosterone, FSH, LH, estradiol, progesterone, or SHBG were observed between the ADHD and control groups for either boys or girls. Among boys with ADHD, urinary levels of monobenzyl phthalate and monoethylhexyl phthalate were positively correlated with serum testosterone levels. By contrast, among girls with ADHD, urinary levels of monoethyl phthalate were positively correlated with serum levels of LH, testosterone, and free testosterone. Taken together, these findings indicate that exposure to EDCs may adversely affect gonadal hormones and neurodevelopment.^23^

### 2.2 Epigenetics (DNA methylation and microRNAs)

#### 2.2.1 MicroRNAs as predictive biomarkers of ADHD and its underlying mechanism

MicroRNAs (miRNAs) are small, noncoding RNAs that are involved in RNA silencing and the posttranscriptional regulation of gene expression, typically downregulating gene expression in human cells. These molecules presumably play a key role in the development of the central nervous system. In our previous study, we included patients with ADHD and HCs. After collecting white blood cells, we conducted RNA enrichment, next‐generation sequencing (NGS), and quantitative polymerase chain reaction experiments. Among the 13 miRNAs examined, 9 exhibited significant differences in abundance between the ADHD and HC groups. We used a support vector machine, a machine learning algorithm, to develop an ADHD prediction model by using these 13 miRNAs. The model achieved a high area under the receiver operating characteristic curve value of 0.94.^24^

To determine the underlying mechanism linking miRNAs to ADHD, we investigated whether differential gray matter volume is associated with changes in miRNA expression levels in patients with ADHD. The results indicated that the expression of miRNAs, particularly miR‐140‐3p, was correlated with the gray matter volume of the left fusiform gyrus, which is associated with ADHD‐related behavioral symptoms. Structural equation modeling revealed that the effect of miR‐140‐3p on hyperactivity and impulsivity symptoms was mediated by the left fusiform gyrus. Taken together, these findings confirm that miRNA expression levels can influence the development of brain structures and contribute to the pathophysiology of ADHD.^25^

#### 2.2.2 miRNAs promote the differentiation of neuronal cells by repressing apoptosis

Given that miR‐140‐3p, miR‐27a‐3p, miR‐486‐5p, and miR‐151‐5p may serve as therapeutic biomarkers of ADHD,^24^ we further explored the mechanisms underlying the association between ADHD and miRNAs. Specifically, we determined whether miRNAs are associated with gray matter volume and whether they influence neuronal differentiation in an in vitro model. Human cortical neuronal (HCN‐2) cells were transfected with miRNA mimics and subjected to differentiation assays. Differentiation was evaluated using AngioTool, which quantifies the vessel area, vessel length, junction number, and mean lacunarity to assess both endothelial and neuronal cell differentiation. Analysis of the in vitro cell model revealed that miR‐140‐3p and miR‐126‐5p promoted the differentiation of HCN‐2 cells by increasing the length of neurons and the number of junctions. To further explore the underlying pathway, we harvested neuronal cells transfected with scrambled control, miR‐140‐3p, or miR‐126‐5p for 24 h and performed flow cytometry assays. Both microarray and flow cytometry assays confirmed that the observed promotion of neuronal differentiation was mediated by the repression of apoptosis and/or necrosis.^26^ Taken together, these results suggest that miRNA expression levels not only serve as potential biomarkers of ADHD but are also involved in the neurodevelopmental and neuroprotective mechanisms that underlie the pathophysiology of ADHD.

#### 2.2.3 miRNAs as therapeutic biomarkers of ADHD

In a cohort of 92 patients with ADHD who received MPH treatment and completed a 12‐month follow‐up, we examined whether changes in miRNA levels were associated with treatment response and clinical outcomes. The results revealed that miR‐140‐3p, miR‐27a‐3p, miR‐486‐5p, and miR‐151‐5p exhibited differential trends in ΔCt values between treatment responders and nonresponders. Among responders, improvements in ADHD symptoms during the 12‐month follow‐up period were positively correlated with increased expression levels of these miRNAs. Taken together, these findings indicate that the expression levels of miRNAs may serve as both diagnostic and therapeutic biomarkers of ADHD.^26^

#### 2.2.4 DNA methylation

DNA methylation is associated with inflammatory responses and neurodevelopment. Given that ADHD is a common neurodevelopmental disorder, in this study, we used methylation microarray and pyrosequencing techniques to explore potential peripheral blood DNA methylation markers of ADHD. DNA methylation profiling data obtained from the microarray assays revealed several differentially methylated CpG sites between 12 patients with ADHD and 9 controls. Five candidate CpG sites (i.e., cg00446123, cg20513976, cg07922513, cg17096979, and cg02506324) in four genes (*LIME1*, *KCNAB2*, *CAPN9*, and *SPTBN2*) were further analyzed using pyrosequencing. In addition, patient attention was tested using Conners' CPT. After categorizing the participants depending on their CPT performance, we discovered that the DNA methylation levels at two CpG sites in *LIME1* (cg00446123 and cg20513976) were significantly higher in children with low CPT performance, whereas the DNA methylation level at one CpG site in *SPTBN2* (cg02506324) was significantly lower in these children. Taken together, these findings indicate that DNA methylation of the CpG sites in *LIME1* and *SPTBN2* may be associated with attention deficits in children with ADHD. They also indicate that DNA methylation biomarkers, particularly those in *LIME1* and *SPTBN2*, may serve as valuable tools for identifying attention deficits in children in clinical settings.^27^

### 2.3 Gut microbial community

#### 2.3.1 Dysbiosis in the gut microbial community in ADHD

The concept of the “gut–brain axis,” which refers to a link between gut microbiota and brain function, has been increasingly implicated in neuropsychiatric disorders, including ADHD. Our research team was among the first to examine the role of gut microbiota dysbiosis and dietary habits in the pathophysiology of ADHD. We collected fecal samples from patients with ADHD and matched controls to examine the composition of their microbiota through 16S rRNA V3V4 amplicon sequencing. Our results, supported by bioinformatics and statistical analyses, revealed that the gut microbiota of patients with ADHD exhibited significantly higher Shannon and Chao indices compared with the controls. In addition, a linear discriminant analysis effect size analysis was conducted to determine the difference in the relative abundance of *Bacteroides coprocola*, *Bacteroides uniformis*, *Bacteroides ovatus*, and *Sutterella stercoricanis* between the ADHD and control groups. In all patients, *S. stercoricanis* demonstrated a significant association with the intake of dairy products, nuts, seeds, legumes, ferritin, and magnesium. Additionally, *B. ovatus* and *S. stercoricanis* demonstrated a positive correlation with the symptoms of ADHD. Taken together, these findings indicate that the composition of the gut microbiome is associated with dietary patterns and may contribute to susceptibility to ADHD.^28^

#### 2.3.2 Gut microbiome imbalance and inflammation

To determine whether immune function influences the association between gut microbiome dysbiosis and ADHD, we explored the correlation between gut microbiome imbalance and inflammation. We measured the plasma levels of 10 cytokines, namely tumor necrosis factor‐alpha (TNF‐α), interleukin‐6 (IL‐6), IL‐1β, IL‐2, IL‐10, IL‐13, IL‐17A, interferon‐alpha 2 (IFN‐α2), IFN‐γ, and monocyte chemoattractant protein‐1, by using a custom‐made sandwich enzyme‐linked immunosorbent assay (Flowmetrix) developed by Luminex. Our results indicated that the plasma levels of TNF‐α were significantly lower in children with ADHD than in the HCs. In the ADHD group, the levels of TNF‐α were negatively correlated with both ADHD symptom severity and gut microbiome diversity. In addition, Phylogenetic Investigation of Communities by Reconstruction of Unobserved States (PICRUSt) revealed functional predictions for microbiota related to the nervous and immune systems. Notably, pathways associated with proximal tubule bicarbonate reclamation and glutamatergic synapses were identified as the most representative pathways in the ADHD group. Additionally, we observed a slightly higher trend in Firmicutes‐to‐Bacteroidetes ratio (F/B ratio) in children with ADHD compared with HCs, suggesting the potential utility of the F/B ratio as a microbial biomarker of ADHD. Taken together, these findings provide valuable insights into the association between gut microbiome dysbiosis and immune dysregulation, which may contribute to the underlying pathophysiology of ADHD.^29^

#### 2.3.3 Fungal mycobiome and intestinal permeability in ADHD

In addition to bacterial communities, the fungal microbiome (referred to as the mycobiota) is a highly diverse group of multicellular eukaryotes that play a key role in gut health and host intestinal permeability. In our previous study, we examined the effect of fungal mycobiome dysbiosis and intestinal permeability on ADHD. Fecal samples were collected from children with ADHD and HCs. After total DNA was extracted, internal transcribed spacer regions were sequenced using high‐throughput NGS. To determine the biological effects of fungal dysbiosis on intestinal epithelial barrier function, an in vitro permeability assay was conducted using a Caco‐2 cell layer model. At the phylum level, the ADHD group exhibited a significantly higher abundance of *Ascomycota* and a lower abundance of *Basidiomycota* compared with the HCs. At the genus level, the abundance of *Candida*, particularly *Candida albicans*, was significantly higher in patients with ADHD than in the HCs. Furthermore, our in vitro cell assay revealed that secretions from *C. albicans* significantly enhanced the permeability of the Caco‐2 cell layer. To the best of our knowledge, the is the first study to identify an association between fungal mycobiome dysbiosis, compromised intestinal permeability, and susceptibility to ADHD.^30^

#### 2.3.4 Gut leakage markers and cognition in ADHD

Intestinal permeability has been implicated in the pathogenesis of ADHD. In this study, we examined the role of gut leakage biomarkers in susceptibility to ADHD by measuring the serum concentrations of zonulin, occludin, and defensin (DEFA1). Visual attention was evaluated using Conners' CPT, and ADHD core symptoms were rated by parents and teachers through the Swanson, Nolan, and Pelham—Version IV Scale (SNAP‐IV). The results revealed significantly lower levels of DEFA1 in the ADHD group than in the HCs. However, no significant between‐group difference was observed in the serum levels of zonulin and occludin. Notably, the serum levels of DEFA1 were inversely correlated with inattention scores on the SNAP‐IV parent and teacher forms and with hyperactivity/impulsivity scores on the SNAP‐IV teacher form. By contrast, the serum levels of occludin were positively correlated with the detectability subtest of the CPT. Taken together, these findings provide novel insights into the relationship between gut leakage markers and cognitive function, advancing the understanding of the pathophysiology of ADHD.^31^

#### 2.3.5 Potential effect of *Bifidobacterium bifidum* (Bf‐688) supplementation on ADHD

In our previous study, we argued that the gut microbiome may influence early human brain development and play a role in the pathophysiology of ADHD.^28^, ^32^ This insight has spurred interest in manipulating the gut microbiome through probiotics as a potential therapeutic strategy for ADHD.^33^ Psychobiotics, a subset of probiotics, are believed to exert their effects through neurotransmitters such as dopamine, epinephrine, noradrenaline, and 5‐hydroxytryptamine.^34^ In our previous study, we conducted a 12‐week randomized, double‐blind, placebo‐controlled clinical trial to determine the potential effect of *Bifidobacterium bifidum* (Bf‐688) supplementation on ADHD.^35^ The study included children with ADHD who were receiving a stable dose of MPH. These children were randomly assigned to receive either add‐on Bf‐688 (daily bacterial count of 5 × 10^9^ CFUs) or a placebo for 12 weeks. The results indicated that the Bf‐688 group exhibited significant improvements in omission errors and hit reaction time in both the CPT and Conners' Continuous Auditory Test of Attention (CATA), although no such changes were observed in the placebo group. Analysis of the gut microbiome revealed a significant increase in the F/B ratio in the Bf‐688 group only. In addition, we identified a significant negative correlation between N‐glycan biosynthesis and hit reaction time in both the CPT and CATA. Taken together, these findings indicate that probiotic Bf‐688 supplementation can enhance the neuropsychological performance of children with ADHD, presumably by altering the composition of the gut microbiota, ultimately leading to reduced N‐glycan biosynthesis.

## 3 CONCLUSION AND FUTURE DIRECTIONS

In this review, we discussed various biomarkers with potential applicability in the diagnosis and treatment of ADHD, emphasizing the need for biomarkers that are noninvasive, reliable, reproducible, and cost‐effective. We examined biomarkers derived from saliva, blood, and stools, representing promising candidates that may serve as objective diagnostic tools and therapeutic targets for ADHD. In addition, we reviewed the role of the neuroendocrine system, focusing on sex‐related hormones and their associated genetic polymorphisms as well as appetite‐regulating hormones. Moreover, we examined the effect of EDCs on ADHD susceptibility. We explored peripheral blood markers, including DNA methylation and miRNAs, in the context of ADHD epigenetics. By integrating clinical data, brain imaging findings, and basic research involving cell‐line studies, we developed a diagnostic model based on miRNAs and demonstrated the involvement of miRNAs in neurodevelopment and neuroprotection mechanisms. In addition, we identified the therapeutic potential of miRNA expression levels in ADHD. To explore the concept of the gut–brain axis, we analyzed fecal samples and found evidence linking the gut microbiome community to dietary patterns and susceptibility to ADHD, with inflammation serving as a potential mediator. Additionally, we conducted a clinical trial and demonstrated that probiotic Bf‐688 supplementation can enhance neuropsychological performance in children with ADHD, potentially by changing the composition of the gut microbiota. Although this review is not exhaustive, it offers a foundation for designing future clinical studies and trials aimed at confirming the validity of these potential biomarkers. Future studies with larger sample sizes and longer follow‐up durations are warranted to confirm these biomarkers for ADHD.

## CONFLICT OF INTEREST STATEMENT

The authors declare no conflict of interest.

## Data Availability

The data that support the findings of this study are available on request from the corresponding author. The data are not publicly available due to privacy or ethical restrictions.

## References

[1] Franke B , Michelini G , Asherson P , Banaschewski T , Bilbow A , Buitelaar JK , et al. Live fast, die young? A review on the developmental trajectories of ADHD across the lifespan. Eur Neuropsychopharmacol. 2018;28(10):1059–1088. 30195575 10.1016/j.euroneuro.2018.08.001 PMC6379245

[2] Johnson MH , Gliga T , Jones E , Charman T . Annual research review: infant development, autism, and ADHD—early pathways to emerging disorders. J Child Psychol Psychiatry. 2015;56(3):228–247. 25266278 10.1111/jcpp.12328

[3] American Psychiatric Association . Diagnostic and statistical manual of mental disorders, fifth edition, Text Revision (DSM‐5‐TR™). Washington, DC: Amer Psychiatric Pub Inc; 2022.

[4] Faraone SV , Bonvicini C , Scassellati C . Biomarkers in the diagnosis of ADHD—promising directions. Curr Psychiatry Rep. 2014;16(11):497. 25298126 10.1007/s11920-014-0497-1

[5] Scassellati C , Bonvicini C , Faraone SV , Gennarelli M . Biomarkers and attention‐deficit/hyperactivity disorder: a systematic review and meta‐analyses. J Am Acad Child Adolesc Psychiatry. 2012;51(10):1003–1019 e20. 23021477 10.1016/j.jaac.2012.08.015

[6] Thome J , Ehlis AC , Fallgatter AJ , Krauel K , Lange KW , Riederer P , et al. Biomarkers for attention‐deficit/hyperactivity disorder (ADHD). A consensus report of the WFSBP task force on biological markers and the world federation of ADHD. World J Biol Psychiatry. 2012;13(5):379–400. 22834452 10.3109/15622975.2012.690535

[7] Polanczyk GV , Willcutt EG , Salum GA , Kieling C , Rohde LA . ADHD prevalence estimates across three decades: an updated systematic review and meta‐regression analysis. Int J Epidemiol. 2014;43(2):434–442. 24464188 10.1093/ije/dyt261 PMC4817588

[8] Rucklidge JJ . Gender differences in attention‐deficit/hyperactivity disorder. Psychiatr Clin North Am. 2010;33(2):357–373. 20385342 10.1016/j.psc.2010.01.006

[9] Davies W . Sex differences in attention deficit hyperactivity disorder: candidate genetic and endocrine mechanisms. Front Neuroendocrinol. 2014;35(3):331–346. 24680800 10.1016/j.yfrne.2014.03.003

[10] Maninger N , Wolkowitz OM , Reus VI , Epel ES , Mellon SH . Neurobiological and neuropsychiatric effects of dehydroepiandrosterone (DHEA) and DHEA sulfate (DHEAS). Front Neuroendocrinol. 2009;30(1):65–91. 19063914 10.1016/j.yfrne.2008.11.002 PMC2725024

[11] Perez‐Neri I , Montes S , Ojeda‐Lopez C , Ramirez‐Bermudez J , Rios C . Modulation of neurotransmitter systems by dehydroepiandrosterone and dehydroepiandrosterone sulfate: mechanism of action and relevance to psychiatric disorders. Prog Neuropsychopharmacol Biol Psychiatry. 2008;32(5):1118–1130. 18280022 10.1016/j.pnpbp.2007.12.001

[12] Wang LJ , Hsiao CC , Huang YS , Chiang YL , Ree SC , Chen YC , et al. Association of salivary dehydroepiandrosterone levels and symptoms in patients with attention deficit hyperactivity disorder during six months of treatment with methylphenidate. Psychoneuroendocrinology. 2011;36(8):1209–1216. 21411231 10.1016/j.psyneuen.2011.02.014

[13] Idkowiak J , Taylor AE , Subtil S , O'Neil DM , Vijzelaar R , Dias RP , et al. Steroid sulfatase deficiency and androgen activation before and after puberty. J Clin Endocrinol Metab. 2016;101(6):2545–2553. 27003302 10.1210/jc.2015-4101 PMC4891801

[14] Reed MJ , Purohit A , Woo LW , Newman SP , Potter BV . Steroid sulfatase: molecular biology, regulation, and inhibition. Endocr Rev. 2005;26(2):171–202. 15561802 10.1210/er.2004-0003

[15] Kent L , Emerton J , Bhadravathi V , Weisblatt E , Pasco G , Willatt LR , et al. X‐linked ichthyosis (steroid sulfatase deficiency) is associated with increased risk of attention deficit hyperactivity disorder, autism and social communication deficits. J Med Genet. 2008;45(8):519–524. 18413370 10.1136/jmg.2008.057729

[16] Davies W , Humby T , Kong W , Otter T , Burgoyne PS , Wilkinson LS . Converging pharmacological and genetic evidence indicates a role for steroid sulfatase in attention. Biol Psychiatry. 2009;66(4):360–367. 19251250 10.1016/j.biopsych.2009.01.001 PMC2720459

[17] Wang LJ , Chan WC , Chou MC , Chou WJ , Lee MJ , Lee SY , et al. Polymorphisms of STS gene and SULT2A1 gene and neurosteroid levels in Han Chinese boys with attention‐deficit/hyperactivity disorder: an exploratory investigation. Sci Rep. 2017;7:45595. 28367959 10.1038/srep45595 PMC5377367

[18] Wang LJ , Huang YH , Chou WJ , Lee SY , Chang HY , Chen CC , et al. Interrelationships among growth hormone, thyroid function, and endocrine‐disrupting chemicals on the susceptibility to attention‐deficit/hyperactivity disorder. Eur Child Adolesc Psychiatry. 2023;32(8):1391–1401. 35119524 10.1007/s00787-021-01886-4

[19] Wang LJ , Huang YH , Chou WJ , Lee SY , Tsai CS , Lee MJ , et al. Potential disturbance of methylphenidate of gonadal hormones or pubescent development in patients with attention‐deficit/hyperactivity disorder: a twelve‐month follow‐up study. Prog Neuropsychopharmacol Biol Psychiatry. 2021;108:110181. 33227299 10.1016/j.pnpbp.2020.110181

[20] Misiak B , Kowalski K , Stanczykiewicz B , Bartoli F , Carra G , Samochowiec J , et al. Appetite‐regulating hormones in bipolar disorder: a systematic review and meta‐analysis. Front Neuroendocrinol. 2022;67:101013. 35792198 10.1016/j.yfrne.2022.101013

[21] Kousi C , Lampri E , Voulgaris S , Vougiouklakis T , Galani V , Mitselou A . Expression of orexin‐A (hypocretin‐A) in the hypothalamus after traumatic brain injury: a postmortem evaluation. Forensic Sci Int. 2021;327:110961. 34454377 10.1016/j.forsciint.2021.110961

[22] Lai KY , Li CJ , Tsai CS , Chou WJ , Huang WT , You HL , et al. Appetite hormones, neuropsychological function and methylphenidate use in children with attention‐deficit/hyperactivity disorder. Psychoneuroendocrinology. 2024;170:107169. 39226626 10.1016/j.psyneuen.2024.107169

[23] Tsai CS , Chou WJ , Lee SY , Lee MJ , Chou MC , Wang LJ . Phthalates, Para‐hydroxybenzoic acids, bisphenol‐a, and gonadal Hormones' effects on susceptibility to attention‐deficit/hyperactivity disorder. Toxics. 2020;8(3):57. 32823738 10.3390/toxics8030057 PMC7560246

[24] Wang LJ , Li SC , Lee MJ , Chou MC , Chou WJ , Lee SY , et al. Blood‐Bourne microRNA biomarker evaluation in attention‐deficit/hyperactivity disorder of Han Chinese individuals: an exploratory study. Front Psychiatry. 2018;9:227. 29896131 10.3389/fpsyt.2018.00227 PMC5987559

[25] Wang LJ , Li SC , Kuo HC , Chou WJ , Lee MJ , Chou MC , et al. Gray matter volume and microRNA levels in patients with attention‐deficit/hyperactivity disorder. Eur Arch Psychiatry Clin Neurosci. 2020;270(8):1037–1045. 31240443 10.1007/s00406-019-01032-x

[26] Wang LJ , Kuo HC , Lee SY , Huang LH , Lin Y , Lin PH , et al. MicroRNAs serve as prediction and treatment‐response biomarkers of attention‐deficit/hyperactivity disorder and promote the differentiation of neuronal cells by repressing the apoptosis pathway. Transl Psychiatry. 2022;12(1):67. 35184133 10.1038/s41398-022-01832-1 PMC8858317

[27] Li SC , Kuo HC , Huang LH , Chou WJ , Lee SY , Chan WC , et al. DNA methylation in LIME1 and SPTBN2 genes is associated with attention deficit in children. Children (Basel). 2021;8(2):92. 33572947 10.3390/children8020092 PMC7912017

[28] Wang LJ , Yang CY , Chou WJ , Lee MJ , Chou MC , Kuo HC , et al. Gut microbiota and dietary patterns in children with attention‐deficit/hyperactivity disorder. Eur Child Adolesc Psychiatry. 2020;29(3):287–297. 31119393 10.1007/s00787-019-01352-2

[29] Wang LJ , Li SC , Li SW , Kuo HC , Lee SY , Huang LH , et al. Gut microbiota and plasma cytokine levels in patients with attention‐deficit/hyperactivity disorder. Transl Psychiatry. 2022;12(1):76. 35197458 10.1038/s41398-022-01844-x PMC8866486

[30] Wang LJ , Li SC , Yeh YM , Lee SY , Kuo HC , Yang CY . Gut mycobiome dysbiosis and its impact on intestinal permeability in attention‐deficit/hyperactivity disorder. J Child Psychol Psychiatry. 2023;64(9):1280–1291. 37016804 10.1111/jcpp.13779

[31] Lee SY , Li SC , Yang CY , Kuo HC , Chou WJ , Wang LJ . Gut leakage markers and cognitive functions in patients with attention‐deficit/hyperactivity disorder. Children (Basel). 2023;10(3):513. 36980071 10.3390/children10030513 PMC10047799

[32] Dash S , Syed YA , Khan MR . Understanding the role of the gut microbiome in brain development and its association with neurodevelopmental psychiatric disorders. Front Cell Dev Biol. 2022;10:880544. 35493075 10.3389/fcell.2022.880544 PMC9048050

[33] Ligezka AN , Sonmez AI , Corral‐Frias MP , Golebiowski R , Lynch B , Croarkin PE , et al. A systematic review of microbiome changes and impact of probiotic supplementation in children and adolescents with neuropsychiatric disorders. Prog Neuropsychopharmacol Biol Psychiatry. 2021;108:110187. 33271210 10.1016/j.pnpbp.2020.110187 PMC8138744

[34] Cocean AM , Vodnar DC . Exploring the gut‐brain axis: potential therapeutic impact of psychobiotics on mental health. Prog Neuropsychopharmacol Biol Psychiatry. 2024;134:111073. 38914414 10.1016/j.pnpbp.2024.111073

[35] Wang LJ , Tsai CS , Chou WJ , Kuo HC , Huang YH , Lee SY , et al. Add‐on *Bifidobacterium Bifidum* supplement in children with attention‐deficit/hyperactivity disorder: a 12‐week randomized double‐blind placebo‐controlled clinical trial. Nutrients. 2024;16(14):2260. 39064703 10.3390/nu16142260 PMC11279422

